# Gastroenteritis in sentinel general practices,The Netherlands.

**DOI:** 10.3201/eid0701.010113

**Published:** 2001

**Authors:** M A de Wit, M P Koopmans, L M Kortbeek, N J van Leeuwen, A I Bartelds, Y T van Duynhoven

**Affiliations:** National Institute of Public Health and the Environment, Department of Infectious Diseases Epidemiology, PO Box 1, 3720 BA Bilthoven, The Netherlands. matty.de.wit@rivm.nl

## Abstract

From 1996 to 1999, the incidence of gastroenteritis in general practices and the role of a broad range of pathogens in the Netherlands were studied. All patients with gastroenteritis who had visited a general practitioner were reported. All patients who had visited a general practitioner for gastroenteritis (cases) and an equal number of patients visiting for nongastrointestinal symptoms (controls) were invited to participate in a case-control study. The incidence of gastroenteritis was 79.7 per 10,000 person years. Campylobacter was detected most frequently (10% of cases), followed by Giardia lamblia (5%), rotavirus (5%), Norwalk-like viruses (5%) and Salmonella (4%). Our study found that in the Netherlands (population 15.6 million), an estimated 128,000 persons each year consult their general practitioner for gastroenteritis, slightly less than in a comparable study in 1992 to 1993. A pathogen could be detected in almost 40% of patients (bacteria 16%, viruses 15%, parasites 8%).


### Gastroenteritis in Sentinel General Practices, the Netherlands

**Matty A.S. de Wit,* Marion P.G. Koopmans,* Laetitia M. Kortbeek,* Nan J. van Leeuwen,* A.I.M. Bartelds,† and Yvonne T.H.P. van Duynhoven***
*National Institute of Public Health and the Environment, Bilthoven, The Netherlands; †Netherlands Institute of Primary Health Care, Utrecht, the Netherlands

From 1996 to 1999, the incidence of gastroenteritis in general practices and the role of a broad range of pathogens in the Netherlands were studied. All patients with gastroenteritis who had visited a general practitioner were reported. All patients who had visited a general practitioner for gastroenteritis (cases) and an equal number of patients visiting for nongastrointestinal symptoms (controls) were invited to participate in a case-control study. The incidence of gastroenteritis was 79.7 per 10,000 person years. *Campylobacter* was detected most frequently (10% of cases), followed by *Giardia lamblia* (5%), rotavirus (5%), Norwalk-like viruses (5%) and *Salmonella* (4%). Our study found that in the Netherlands (population 15.6 million), an estimated 128,000 persons each year consult their general practitioner for gastroenteritis, slightly less than in a comparable study in 1992 to 1993. A pathogen could be detected in almost 40% of patients (bacteria 16%, viruses 15%, parasites 8%).

In industrialized countries, gastroenteritis incidence remains high, although improved hygiene and treatment have decreased deaths substantially (1,2). Up-to-date estimates of the incidence, disease burden (absence from work or school, bed rest, and use of medication), microbiologic causes, transmission routes, and pathogen sources are necessary to control gastroenteritis effectively and evaluate preventive measures. While numerous studies have addressed the incidence of specific pathogens in selected populations, little is known about the overall incidence and relative contribution of the broad range of pathogens that cause gastroenteritis. In the Netherlands, data on the incidence of gastroenteritis are available from studies in the early 1990s in the general population and in general practices and from continuous laboratory-based surveillance (3,4). The incidence in the general population was estimated to be 450 per 1,000 person years in 1991 (5,6). Patients were tested for *Salmonella* and *Campylobacter* spp., which were detected in 1.5% and 4.6%, respectively. Of these patients, 10% reported visiting their general practitioner (GP), and an additional 10% reported phoning their GP. The incidence of gastroenteritis for which a GP was consulted was estimated to be 15 per 1,000 person years in two regions from 1987 to 1991 (7) and 9 per 1,000 person years in 1992-1993 (8). In these two studies, stool samples were tested for bacterial pathogens; a small subset of these were tested for rotavirus and adenovirus. In most patients (67% in 1987 to 1991, 80% in 1992 to 1993), no pathogen could be detected.

In the years following these studies, new pathogens have been identified, such as *Cyclospora*, and diagnostic methods have been designed for others (Norwalk-like viruses, astrovirus). In addition, to meet the European regulations on protection against zoonotic agents, preventive measures in the veterinary sector have been implemented to decrease *Salmonella* infections in poultry (9-11). Therefore, in 1996, a new study was started among patients consulting a GP. The aims were to estimate the current incidence, study the role of several pathogens, and identify risk factors for

Address for correspondence: Matty de Wit, National Institute of Public Health and the Environment, Department of Infectious Diseases Epidemiology, PO Box 1, 3720 BA Bilthoven, the Netherlands; fax: 31-30-274-4409; e-mail: matty.de.wit@rivm.nl.

microorganism-specific gastroenteritis. Unique to this study, controls were included and stool samples were tested for a broad panel of pathogens. The same network of GPs was used as in the 1992-1993 study, allowing a comparative analysis.

### Materials and Methods

The study was performed in cooperation with the network of GPs from the Continuous Morbidity Registration of the Netherlands Institute of Primary Health Care. The network consists of approximately 44 practices that cover 1% of the Dutch population and are representative of it regarding age, gender, regional distribution, and degree of urbanization. All practices reported the number of consultations for gastroenteritis by age group, gender, practice, and week of consultation (reporting study).

Approximately 34 of the practices (33 in 1996; 35 in 1997; 36 in 1998; and 34 in 1999) participated in the case-control study. Patients who consulted a participating GP were asked to complete a questionnaire and submit a stool sample. For each case, a control was recruited on the same day from patients who consulted their GP for nongastrointestinal complaints. Controls were matched by age group ($\leq$11 years, 12 years and older). Thirty-one GPs registered the number of the study package (containing a questionnaire and a stool kit), which was handed out to a participant, on a registration form with age and gender. Participants collected the stool samples and completed the questionnaire at home, and then sent the samples and questionnaires directly to the National Institute of Public Health and the Environment. The questionnaire addressed health status, demographic characteristics, disease burden, clinical manifestations, and risk factors. Stool samples were tested for *Salmonella, Campylobacter, Shigella,* and *Yersinia* by routine culture; for verocytotoxin-producing *Escherichia coli* by polymerase chain reaction for genes for verocytotoxin 1 and verocytotoxin 2 and *eae* gene; *E. coli* O157 by culture on Sorbitol McConkey Agar (12,13); rotavirus and adenovirus by enzyme-linked immunoassay (ELISA) (Rotaclone and Adenoclone from Meridian Diagnostics, Cincinnati, OH); astrovirus by ELISA (IDEIA from DAKO Diagnostics, Cambridgeshire, UK); Norwalk-like viruses by reverse transcriptase-polymerase chain reaction [14]; intestinal parasites by microscopy after fixation in sodium acetate, acetic acid, and formalin; helminthic ova, cysts, and trophozoites by wet film (iodine stained or unstained); for cysts by Ridley concentration (iodine stained); for cysts of *Cryptosporidium*, *Cyclospora*, and *Isospora belli*, by Ridley concentration (Ziehl-Nielsen staining); for trophozoites of protozoa and, to a lesser extent, for cysts, by permanent stained smear (Haemaluin staining) (15-20). Samples from May 1998 until April 1999 were also tested retrospectively for Sapporo-like viruses (21). All stool samples were stored for future investigations. Standardized protocols were used.

The case definition of gastroenteritis used in this study was three or more loose stools in 24 hours; or diarrhea with two additional gastrointestinal symptoms (vomiting, nausea, fever, abdominal cramps, abdominal pain, blood in stool, mucus in stool); or vomiting with two additional gastrointestinal symptoms (diarrhea, nausea, fever, abdominal cramps, abdominal pain, blood in stool, mucus in stool) preceded by a symptom-free period of 2 weeks.

### Statistical Analyses

Crude incidence was calculated from the reporting study, corrected for incomplete years. Corrections for incompleteness of participation of GPs and cases and for list inflation (persons no longer belonging to the practice population) were used to calculate an adjusted incidence. List inflation was calculated on the basis of findings in an ongoing, population-based study, performed in the same general practices (22).

The response rate in cases and controls was estimated by using the registration forms. Reported cases and cases in the case-control study were compared by week, practice, age group, and gender to estimate the number of cases in both and each study part. The total cases reported, recruited for the case-control study, or both were used to estimate an incidence corrected for nonresponse. A logistic regression model was used to identify factors independently associated with total response. A variable was excluded if exclusion did not significantly decrease the log likelihood of the model. Because information on the response of recruited cases was too limited to study effects of patients and practice characteristics, only the total of known cases to participating cases was studied in this model.

The effects on incidence of age and gender of the patient, year of study, degree of urbanization, region of the Netherlands, and participation of the sentinel practice in the case-control study were estimated univariately; independent effects were estimated by using Poisson regression. A variable was excluded from the model if exclusion did not increase the deviance significantly. Cases that did not meet the case definition and controls that did, according to self-reported symptoms in the questionnaire, were excluded from analyses.

### Results

The overall incidence of gastroenteritis from May 1996 to April 1999 was 58.0 per 10,000 person years (2,264 cases/390,417 person years) based on the unadjusted number of reported patients of all sentinel practices. A high seasonal peak was observed in the winter of 1996 (maximum in week 9), and lower peaks in 1998 and 1999 (Figure). Summer peaks occurred in all years of the study but varied in period and size.

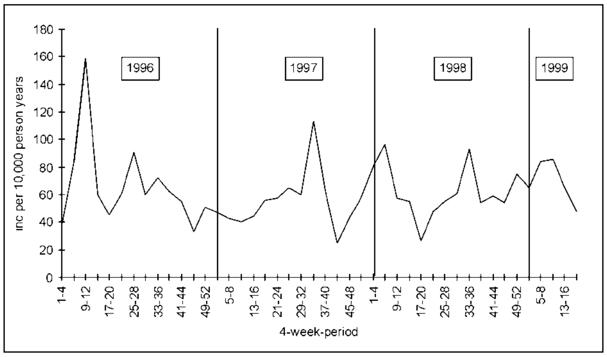

Figure. Incidence of gastroenteritis per 10,000 person years, from reporting of all sentinel practices, the Netherlands, January 1996 to April 1999.

The univariate and multivariate analyses of incidence yielded similar results (Table 1). In the first year of the study (May 1996-April 1997), incidence was lower than in the second (May 1997-April 1998) and third years (May 1998-April 1999). A higher incidence was observed in practices that participated in the case-control study, practices in rural and urban areas (compared to those with intermediate degree of urbanization), and practices in the East, West, and South. The incidence was slightly higher for women than for men and decreased from the youngest age group to the 15- to 24-year age group. The 40- to 64-year-old patients had the lowest incidence. The rate ratios for the different age groups were similar for men and women.

The response of patients was 78% (695 questionnaires completed out of 888 cases registered on registration forms). A total of 2,553 cases were reported, recruited for the case-control study, or both. Of these, 1,138 (45%) patients were recruited, 888 (35%) participated in the case-control study, and 2,165 (85%) were reported. The incidence corrected for incomplete participation of patients and GPs was 77.7 per 10,000 person years (2,553 cases/328,438 person years), according to data from GPs participating in the case-control study. The estimated list inflation was 2.5%. Adjusting the denominator for list inflation yielded a final incidence of 79.7 per 10,000 person years (2,553 cases/320,227 person years).

The percentage of patients recruited by GPs decreased during the study and was higher in the North and South (Table 2). The highest participation rate was for patients 25-64 years of age, mainly because a higher proportion were recruited. Participation was relatively low for 15- to 24-year-old patients.

The response of controls who were recruited was 73% (554 questionnaires completed out of 765 study packages registered). The lowest response rates for controls (42%) were found in the 10- to 19-year-old age group. The response for female controls was slightly higher than for male controls (78% versus 68%). Participation was highest for controls recruited in 1996 (82%) and lowest in 1999 (63%).

### Case-Control Study

### Population of Participating Practices

The distribution of age, gender, and degree of urbanization in the study population was similar to that of the Dutch population. The North was overrepresented (20% in participating practices, 10% in Dutch population); the West was underrepresented (32% in practices vs. 44% in the population).

### Cases and Controls

From May 1996 until April 1999, 985 cases and 717 controls returned a questionnaire to the National Institute of Public Health and the Environment. Of these, 878 cases and 581 controls could be included in the analyses. Stool samples were examined from 857 (98%) patients and 574 (99%) controls. The median age of patients (29 years) was significantly lower than

Table 1. Univariate and multivariate Poisson regression analyses of the incidence per 10,000 person years

| Incidence per 10,000 person years | Crude incidence | Univariate Poisson regression | | Multivariate Poisson regression | |
|---|---|---|---|---|---|
| | | RR[a] | 95% CI[a] | RR | 95% CI |
| Study year | | | | | |
| May 96-Apr 97 | 52.4 | 1.00 | - | 1.00 | - |
| May 97-Apr 98 | 62.4 | 1.19 | 1.08-1.32 | 1.19 | 1.08-1.32 |
| May 98-Apr 99 | 65.6 | 1.25 | 1.13-1.38 | 1.23 | 1.11-1.36 |
| Participation in case-control study | | | | | |
| Yes | 65.8 | 2.51 | 2.13-2.97 | 2.71 | 2.28-3.22 |
| No | 26.2 | 1.00 | - | 1.00 | - |
| Urbanization | | | | | |
| Low | 57.0 | 1.04 | 0.92-1.17 | 1.18 | 1.04-1.34 |
| Intermediate | 55.5 | 1.00 | - | 1.00 | - |
| High | 88.9 | 1.62 | 1.47-1.79 | 1.25 | 1.11-1.41 |
| Region | | | | | |
| North | 28.7 | 0.39 | 0.34-0.45 | 0.35 | 0.29-0.40 |
| East | 71.3 | 0.97 | 0.88-1.07 | 0.93 | 0.83-1.05 |
| West | 76.0 | 1.00 | - | 1.00 | - |
| South | 55.5 | 0.75 | 0.68-0.84 | 0.78 | 0.69-0.88 |
| Gender | | | | | |
| Male | 56.8 | 1.00 | - | 1.00 | - |
| Female | 63.4 | 1.12 | 1.03-1.21 | 1.12 | 1.04-1.22 |
| Age (in years) | | | | | |
| <1 | 360.4 | 5.53 | 4.49-6.82 | 5.54 | 4.50-6.83 |
| 1-4 | 221.5 | 3.40 | 2.94-3.94 | 3.36 | 2.90-3.89 |
| 5-14 | 65.1 | 1.00 | - | 1.00 | - |
| 15-24 | 51.7 | 0.79 | 0.67-0.94 | 0.79 | 0.67-0.93 |
| 25-39 | 55.9 | 0.86 | 0.75-0.98 | 0.84 | 0.73-0.97 |
| 40-64 | 36.5 | 0.56 | 0.48-0.65 | 0.56 | 0.48-0.65 |
| ≥65 | 47.7 | 0.73 | 0.62-0.87 | 0.72 | 0.61-0.85 |

[a]RR = relative risk; CI = confidence interval.

that of controls (37 years). Gastrointestinal complaints of longer than 1 month were more frequent in patients (31.5%) than in controls (7.3%). The percentage of patients (18%) under treatment of a specialist was similar to that of controls, as was the number of GP consultations in the last 3 months (1 consultation). Patients were slightly more often born outside the Netherlands than controls, and the educational level of patients was higher than that of controls (both not statistically significantly).

### Clinical Symptoms of Patients

Almost all patients reported loose stools (Table 3). Other commonly reported symptoms were frequent stools, abdominal pain, abdominal cramps, and nausea. Only 12 (1.4%) patients reported vomiting and no diarrhea. In cases with a higher frequency of stools than normal, the median of the maximum frequency was 6 times in 24 hours (first quartile-third quartile: 4-8 times in 24 hours). The median duration of symptoms before a GP was consulted was 6 days (first quartile to third quartile: 3-20 days); 20% of patients had been symptomatic for more than 4 weeks before consulting a GP.

Bed rest was required for 47% of patients for a median of 2 days (mean 3.1 days); 41% of the children who regularly visited a day-care center had to stay home for a median of 3 days (mean 3.2 days), 58% of schoolchildren were absent from school for a median duration of 3 days (mean 3.8 days), and 60% of working patients were absent from work for a median duration of 2 days (mean 3.1 days). In 8% of cases, someone had to miss school or work for a median of 1.5 days (average 2.0 days) to care for the patient; 56% of patients used medication for gastroenteritis: 4% used antibiotics, 18% analgesics, 27% antidiarrheic medication, 10% oral rehydration solution, and 21% additional medications.

### Symptoms and Diagnoses of Controls

A small proportion of controls (6.7%) reported consulting a GP for gastrointestinal symptoms when they were recruited for the study but did

Table 2. Multivariate logistic regression analyses of the selection of participating cases from all cases that were reported or participated in the case-control study, the Netherlands

| | Recruited or reported | % Recruited by GP[a] | % Part. cases[b] | % Total response | OR[c] for response | 95% CI[d] |
|---|---|---|---|---|---|---|
| Gender | | | | | ni[e] | |
| Male | 1,178 | 45 | 79 | 35 | | |
| Female | 1,370 | 44 | 78 | 35 | | |
| Age (yrs) | | | | | | |
| 0 | 123 | 34 | 76 | 26 | 0.84 | 0.51-1.37 |
| 1-4 | 452 | 40 | 76 | 31 | 0.89 | 0.63-1.25 |
| 5-14 | 342 | 36 | 82 | 30 | 0.85 | 0.59-1.21 |
| 15-24 | 265 | 51 | 65 | 33 | 1.00 | - |
| 25-39 | 621 | 51 | 76 | 39 | 1.30 | 0.95-1.78 |
| 40-64 | 487 | 50 | 85 | 42 | 1.50 | 1.08-2.07 |
| 65+ | 254 | 35 | 92 | 32 | 0.89 | 0.61-1.30 |
| Urbaniztion | | | | | ni | |
| Low | 305 | 34 | 94 | 32 | | |
| Intermediate | 1,654 | 46 | 79 | 37 | | |
| High | 582 | 45 | 72 | 32 | | |
| Region | | | | | | |
| North | 281 | 60 | 83 | 49 | 2.16 | 1.63-2.87 |
| East | 586 | 33 | 84 | 28 | 0.86 | 0.68-1.08 |
| West | 1,034 | 41 | 73 | 30 | 1.00 | - |
| South | 642 | 53 | 81 | 43 | 1.69 | 1.36-2.08 |
| Year of study | | | | | | |
| May 96-Apr 97 | 804 | 59 | 80 | 47 | 1.00 | |
| May 97-Apr 98 | 841 | 40 | 81 | 32 | 0.50 | 0.43-0.65 |
| May 98-Apr 99 | 889 | 34 | 77 | 26 | 0.40 | 0.33-0.50 |
| Total | 2,553 | 45 | 78 | 35 | - | - |

[a]GP - general practitioner
[b]part = participating.
[c]OR = odds ratio for participation (defined as a case questionnaire received at the National Institute of Public Health and the Environment).
[d]CI = confidence interval.
[e]ni = not included in the logistic regression model.

Table 3. Self-reported symptoms of patients, Netherlands case-control study

| | No. of cases (N=878) | % of cases |
|---|---|---|
| Loose stools | 861 | 98.1 |
| Frequent stools[a] | 387 | 78.2 |
| Abdominal cramps | 679 | 77.3 |
| Abdominal pain | 673 | 76.7 |
| Nausea | 536 | 61.0 |
| Vomiting | 359 | 40.9 |
| Fever | 335 | 38.2 |
| Mucus in stool | 304 | 34.6 |
| Blood in stool | 97 | 11.0 |

[a]More frequent than normal as perceived by the respondent.

not meet the case definition; 14% consulted for other reasons (e.g., to pick up a prescription, routine physical examination, accompanying a relative); most controls gave no information (43%) or had consultations for other symptoms (36%).

### Pathogens

The most frequently detected pathogens in cases were *Campylobacter*, *Giardia lamblia*, rotavirus, Norwalk-like viruses, and *Salmonella* (Table 4). Bacterial pathogens were found almost solely in cases, except for *Yersinia* and verocytotoxin-producing *E. coli*. All isolated *Yersinia* spp. were nonpathogenic serotypes. The verocytotoxin-producing *E. coli* serotypes found in controls were different from those found in patients but included pathogenic types, such as O26. *E. coli* O157 K-H- was isolated from one case. The percentages of *Campylobacter, Salmonella,* and *Salmonella* Enteritidis did not differ significantly over the study years. Viral pathogens were found in 1% to 5% of patients and in a small percentage of controls. The possibly nonpathogenic parasite *Dientamoeba fragilis* and the nonpathogenic parasite *Blastocystis hominis* were common and were found more frequently in controls than in patients.

Table 4. Microorganisms detected in patients and controls

| | Patients (N=857) | | Controls (N=574) | |
|---|---|---|---|---|
| | N | % | N | % |
| *Salmonella* spp. | 33 | 3.9 | 1 | 0.2 |
| *S.* Enteritidis | 12 | 1.4 | 0 | 0.0 |
| *S.* Typhimurium | 11 | 1.3 | 1 | 0.2 |
| other *Salmonellae*[a] | 9 | 1.1 | 0 | 0.0 |
| *Campylobacter* spp. | 89 | 10.5 | 3 | 0.5 |
| *C. jejuni* | 77 | 9 | 3 | 0.5 |
| *C. coli* | 7 | 0.8 | 0 | 0.0 |
| other species | 5 | 0.6 | 0 | 0.0 |
| *Yersinia* spp. | 6 | 0.7 | 6 | 1.1 |
| *Y. enterocolitica*[b] | 5 | 0.6 | 5 | 0.9 |
| *Y. frederiksenii*[c] | 1 | 0.1 | 1 | 0.2 |
| *Shigella* spp. | 1 | 0.1 | 0 | 0.0 |
| *S. flexneri* | 1 | 0.1 | 0 | 0.0 |
| VTEC[d] | 4 | 0.5 | 3 | 0.6 |
| Rotavirus | 45 | 5.3 | 8 | 1.4 |
| Adenovirus | 19 | 2.2 | 2 | 0.4 |
| Astrovirus | 13 | 1.5 | 2 | 0.4 |
| Norwalk-like viruses | 43 | 5.0 | 6 | 1.1 |
| Sapporo-like viruses[e] | 5 | 2.1 | 1 | 0.6 |
| *Giardia lamblia* | 46 | 5.4 | 19 | 3.3 |
| *Entamoeba histolytica / dispar* | 9 | 1.1 | 4 | 0.7 |
| *Cryptosporidium* | 18 | 2.1 | 1 | 0.2 |
| *Cyclospora* | 1 | 0.1 | 1 | 0.2 |
| (Possibly) nonpathogenic microorganisms | | | | |
| *Dientamoeba fragilis* | 88 | 10.3 | 84 | 14.6 |
| *Blastocystis hominis* | 185 | 21.7 | 172 | 30.0 |
| *Endolimax nana* | 14 | 1.6 | 14 | 2.4 |
| *Enteromonas hominis* | 2 | 0.2 | 1 | 0.2 |
| *Chilomastix mesnili* | 1 | 0.1 | 0 | 0.0 |
| *Entamoeba hartmanni* | 0 | 0.0 | 2 | 0.4 |
| *Enterobius vermicularis* | 0 | 0.0 | 1 | 0.2 |
| *Iodamoeba butschlii* | 0 | 0.0 | 1 | 0.2 |

[a]*S.* Heidelberg, *S.* Thompson, *S.* Kottbus, *S.* Montevideo, *S.* Manhattan, *S.* Anatum, *S.* Arizona, *Salmonella* group B (not fully typeable).
[b]Serotypes of *Y. enterocolitica* in patients: O6,31 (biotype 1a), O6,30; O6,30 (biotype 1A), one not typeable, one not typed. In controls: two O4 (biotype 1A); O65 (biotype 1A), O7,8 (biotype 1A); one not typeable.
[c]Serotypes of *Y. frederiksenii*: O16a,58 (case); O16AB,29 (control).
[d]VTEC=verocytotoxin-producing *Escherichia coli*: positive for *eae* gene and genes for either SLT1 or SLT2. VTEC serotypes in cases: O157 K-H-, O98 K-, O145 K-, one not typeable. In controls: O26, O145 K-, one not typeable
[e]Sapporo-like viruses have only been tested for the last study year (May 98–Apr 99; 240 cases and 160 controls). Genotypes in cases: 4 Sapporo, 1 London, genotype in control: Sapporo.

In 37.5% of patients and 9.8% of controls, a pathogen could be detected (excluding possibly nonpathogenic microorganisms, Table 4). From May 1998 until May 1999, these percentages were increased by Sapporo-like viruses with 1.7% in patients and 0.6% in controls. When *D. fragilis* was considered a pathogen, these percentages were 44.1% and 22.9%, respectively. The percentage in which a pathogen was found was similar for the 50 controls who reported gastrointestinal symptoms (but did not meet the case definition) and controls without gastrointestinal symptoms.

Most stool samples of patients were received at the National Institute within 1 day of collection (57%), 21% after 2 days, 9% after 3 days, 7% after 4 days, 3% after 5 days, and 2% after ≥6 days. No significant differences were noted in the percentage of samples in which a pathogen was detected for different postal delays. For *Campylobacter*, however, a decreasing trend in the proportion of positive cases was observed with an increasing postal delay: 1 day: 12% positive, 2 days: 10%, 3 days: 9%, 4 days: 6%, 5 days 5%, 6 days or more: no *Campylobacter*.

### Discussion

### Incidence and Participation Rate

This is the first GP-based national study in the Netherlands covering the role of a wide range of microorganisms in a representative population of gastroenteritis patients and controls. Only England has conducted a similar study.

The incidence of gastroenteritis in general practices was estimated at 79.7 per 10,000 person years, suggesting that in the Netherlands, 1 in every 125 persons, or 128,000 persons, will seek physician care for gastroenteritis every year. This estimate was adjusted for list inflation and partially for nonparticipation (15%) of GPs but not for the number of underascertained cases absent from both study components. In a similar study in England, underascertainment was estimated to be 36% (23). The correction for list inflation (2.5%) in our study should be considered a minimum estimate because only patients who were actively reported as no longer belonging to the general practice population were counted; by contrast, the more active approach in England (searching medical records of nonrespondents)

yielded an estimate of 10% (23). Consequently, the incidence from our study must be considered a conservative, but the best available, estimate.

A previous estimate of the incidence of gastroenteritis in a GP-based study in 1992 to 1993 in the Netherlands was somewhat higher at 90 cases per 10,000 person years (not corrected for list inflation) (8). Since the percentages of *Salmonella* and *Campylobacter* have also decreased since 1992-1993 (from 5% to 4% and from 14% to 10%, respectively), the decrease in incidence could partially be due to preventive measures in the poultry industry that focused on *Salmonella* Enteritidis, but as expected, also caused a slight decrease in *Campylobacter* infections in poultry. Nevertheless, these measures were not fully implemented until April 1997, and therefore a decreasing trend in the incidence within the study period would be expected, which was not observed for gastroenteritis as a whole, nor for the percentages positive for *Salmonella* and *Campylobacter*. Several other factors might have contributed to this decrease, such as a more widespread use of Hazard Analysis and Critical Control Points in the food production industry, greater awareness and knowledge in the population about foodborne infections, and a change in consultation behavior because of GPs' increasing deferral policy for gastroenteritis.

The low incidence in the first study year was presumably due to the absence of a winter peak in 1996 to 1997. Such peaks coincide with an increase in infections with enteric viruses, such as astrovirus, rotavirus, and calicivirus (24-26). Therefore, the variation in incidence in different years likely is due to the annual fluctuation of the peaks in viral pathogens.

Our data indicate an incidence almost fourfold lower than the incidence in England. The GP-based incidence of gastroenteritis estimated for England in a study from 1993 to 1996 was 330 per 10,000 person years after correction for list inflation and underreporting (27). In Wales in 1992, an incidence of 244 per 10,000 person years was found (28). Most likely, gastroenteritis patients' higher consultation rate in England accounts for the difference: approximately 1 in 6 gastroenteritis case-patients in England consult a GP, whereas an estimated 1 patient in 10 to 1 patient in 50 does in the Netherlands (5,6,8). A lower consultation rate could be explained by Dutch GPs' policy of deferring gastroenteritis cases. In the Dutch Guidelines for Acute Diarrhea, a consultation by telephone is considered adequate for uncomplicated acute diarrhea and can be dealt with by the GP's assistant (29). Of the 19 participating GPs who completed a questionnaire at the end of our study, 12 (63%) reported fully or partially discouraging patients with gastroenteritis from visiting their practice. In England, a deferral policy for consultations for gastroenteritis is not common.

The incidence of gastroenteritis was independently associated with degree of urbanization and geographic region. The incidence was the lowest in the North, as was found in the study in 1992 to 1993 (8). This cannot be explained by a lower response rate (Table 2). A higher incidence was found in areas with a high or low degree of urbanization than in areas with an intermediate degree of urbanization. Other studies also report a higher incidence in urban areas (30). Possibly, a high population density causes an increased risk for person-to-person transmission. In rural areas, contact with animals, manure, and raw products could be more frequent than in more urbanized regions, possibly leading to higher levels of exposure to zoonotic pathogens. An analysis of risk factors for each pathogen will be published after an ongoing population-based study is completed. The differences in incidence could also reflect differences in consultation behavior.

Patient-related factors that were independently associated with incidence were patient's gender and age. The incidence was higher for women than for men and was clearly higher in the youngest age groups, consistent with other studies (5,8,28,31-33). Although several studies have reported a higher disease burden for the elderly, no increase in the incidence of gastroenteritis was observed in our study (1). Possibly, the higher risk for gastroenteritis for the elderly is limited to the relatively weaker persons living in nursing homes (25). Because these homes usually have their own GP, the proportion of persons living in nursing homes could be underrepresented in this study.

### Pathogens

Bacteria were detected in 16.3% of patients, viruses in 15.4%, and pathogenic parasites in

8.3%. The higher participation of the northern region might have increased the percentage of *Salmonella* Typhimurium because *S.* Typhimurium is predominant over *S.* Enteritidis in this region, in contrast to the rest of the Netherlands (34). The order of relative importance of pathogens in general practices was similar for the Netherlands and England, although the percentages positive for viruses and bacteria in this study were slightly lower than in England and the percentages positive for parasites were slightly higher. Because bacteria and viruses are more often detected in patients with acute diarrhea, whereas parasites tend to cause less fulminant but intermittent and long-lasting symptoms that might lead to delayed consultation, these differences could be due to the exclusion of persons with symptoms lasting longer than 2 weeks in the English study (35,36). In our study, 32% of patients had symptoms >2 weeks' duration (36). In addition, we used formalin-fixed material to detect parasites, and four different preparations were examined, which increased the sensitivity of microscopy examination, compared to the use of nonfixed material and the examination of three different preparations in the English study (37). The lower participation of patients in the last 2 years of the study might have caused an underestimate of viral pathogens because the seasonal viral peak was relatively low in the first year (24,38). In spite of a less sensitive method of testing for Norwalk-like viruses (electron microscopy in England versus reverse transcriptase-polymerase chain reaction in the Netherlands), the percentage of stool samples positive for Norwalk-like viruses is higher in England (36). The low response for the younger age groups might also have reduced the percentage of rotavirus, Sapporo-like viruses, and to a lesser degree Norwalk-like viruses, which are most common in young children (24). Differences in the proportion of specific pathogens in the English study and in our study may also be explained by differences in consultation rates. A lower threshold for consulting a GP might increase the proportion of pathogens that cause relatively mild gastroenteritis, such as Norwalk-like viruses.

Parasites that were (possibly) nonpathogenic were more frequently found in controls than in patients. A more detailed study of differences between patients and controls with these parasites might identify factors related to the development of disease after infection with these parasites.

### Diagnostic Deficit

In spite of a diagnostic panel that included most of the known pathogens that can cause gastroenteritis, the percentage of patients in which no pathogen could be detected was 61% (including Sapporo-like viruses in 1998 and excluding *D. fragilis* and nonpathogenic parasites). Some cases could be noninfectious because an exclusive distinction between infectious and noninfectious intestinal disease cannot be made clinically. The high percentage of patients with chronic gastrointestinal symptoms suggests that a substantial proportion might not be infectious but an expression of other illnesses, such as inflammatory bowel disease. The symptoms might also be caused by intestinal microorganisms not included in this study or not yet known. Several pathogens, such as *Campylobacter*, verocytotoxin-producing *E. coli*, and torovirus have only recently been recognized as a cause of gastroenteritis, and it is likely that new pathogens will be added to this list (39). In addition, we did not screen for some pathogens, such as some pathogenic *E. coli* (e.g., enterotoxigenic *E. coli*, enteroinvasive *E. coli*) and bacterial toxins (*Bacillus* spp, *Clostridium difficile* cytotoxin, *C. perfringens* enterotoxin), which were associated with 15% and 6% of the cases, respectively, in the English study (36). However, all stool samples from our study were stored and are currently being tested for Sapporo-like viruses and can be tested for other pathogens. Also, intestinal symptoms can be caused by nonintestinal infections, such as influenza, not included in our study. Approximately one quarter of gastroenteritis cases in an American population-based study coincided with respiratory disease (31). Detection of pathogens in the study is also influenced by logistics factors and the sensitivity of the testing method. The timing of sampling was not ideal in many cases; because of the relatively long patient delay, some pathogens might no longer have been excreted in the stool at the time of sampling. Some pathogens, such as *Campylobacter,* do not grow in culture after a long delay in shipping. At present, we are conducting a study on gastroenteritis in the community to elucidate the role of sampling time and referral behavior on pathogen-specific incidence.

### Acknowledgments

We thank the participating general practitioners and the Netherlands Institute for Primary Health Care for their indispensable cooperation in the data collection; Jan Vinjé for his work on the diagnostic testing methods and support; Joke Admiraal, Denise Hoek, Nahid Nozari, Hanneke Deijl, Petra de Bree, Jeroen Meijer, and Sandy Altena for their excellent assistance in performing the diagnostic tests; Isabel Araya Segovia for administrative support; Martien Borgdorff for his work in the design and start of the study; and Nico Nagelkerke for his advice on data analyses.

Ms. de Wit works as an epidemiologist at the Department of Infectious Disease Epidemiology of the National Institute of Public Health and the Environment in the Netherlands. Her work focuses on the epidemiology of gastroenteritis in the Netherlands at the hospital, general practice, and community levels.

### References

1. Guerrant RL, Hughes JM, Lima NL, Crane J. Diarrhea in developed and developing countries: magnitude, special settings and etiologies. Rev Infect Dis 1990;12:S41-50.
2. Bern C, Martines J, de Zoysa I, Glass RI. The magnitude of the global problem of diarrhoeal disease: a ten-year update. Bull World Health Organ 1992;70:705-14.
3. Esveld MI, van Pelt W, van Leeuwen WJ, Banffer JRJ. Laboratorium Surveillance Infectieziekten. RIVM report no. 968902002. Bilthoven, the Netherlands: RIVM; June 1996.
4. Sprenger MJW, Schrijnemakers PM, Wijgergangs LM, Talsma E. Infectious Diseases Surveillance and Information System (ISIS). International Conference on Emerging Infectious Diseases; 1998 Mar 8-11; Atlanta. p. 123.
5. de Wit MAS, Hoogenboom-Verdegaal AMM, Goosen ESM, Sprenger MJW, Borgdorff MW. A population-based longitudinal study on the incidence and disease burden of gastroenteritis and *Campylobacter* and *Salmonella* infection in four regions of the Netherlands. Eur J Epidemiol 2000;16:713-8.
6. Hoogenboom-Verdegaal AMM, Jong JC, During M, Hoogenveen R, Hoekstra JA. Community-based study of the incidence of gastrointestinal disease in the Netherlands. Epidemiol Infect 1994;112:481-7.
7. Hoogenboom-Verdegaal AMM, Goosen ESM, During M, Engels GB, Klokman-Houweling JM, Laar MJW van de. Epidemiologisch en microbiologisch onderzoek met betrekking tot acute gastro-enteritis in de huisartsenpeilstation in Amsterdam en Helmond, 1987-1991. RIVM report no. 149101011. Bilthoven, the Netherlands: RIVM; 1994.
8. Goosen ESM, Hoogenboom-Verdegaal AMM, Bartelds AIM, Sprenger MJW, Borgdorff MW. Incidentie van gastro-enteritis in huisartsenpeilstations in Nederland, 1992-1993. RIVM report no. 149101012. Bilthoven, the Netherlands: RIVM; 1995.
9. Council directive 92/117/EEC, concerning measures for protection against specified zoonoses and specified zoonotic agents in animals and products of animal origin in order to prevent outbreaks of food-borne infections and intoxications. 1992 Dec. Productschap Vlee, Vleesen Eieren. Rijswijk: PVE; 1997a.
10. Productschap Vee, Vlees en Eieren (PVE). Plan van aanpak preventie en bestrijding Salmonella in de eiersector. Rijswijk, the Netherlands: PVE; 1997a.
11. Productschap Vee, Vlees en Eieren (PVE). Plan van aanpak Salmonella en Campylobacter in de pluimveevleessector. Rijswijk, the Netherlands: PVE; 1997b.
12. Smith HR, Scotland SM. Isolation and identification methods for *Escherichia coli* O157 and other verotoxin producing strains. J Clin Pathol 1993;46;10-17.
13. Edwards and Ewing. Identification of Enterobacteriaceae. 4th ed. New York: Elsevier Science Publishing Co., Inc.; 1986.
14. Vinjé J, Koopmans MPG. Molecular detection and epidemiology of small round-structured viruses in outbreaks of gastroenteritis in the Netherlands. J Infect Dis 1996;174:610-15.
15. Polderman AM, Rijpstra AC, editors. Medische parasitologie, handleiding bij laboratorium diagnostiek. Bohn Stafleu Van Loghum, the Netherlands; Houten/Zaventem; 1993; 218:146-7.
16. Belding DL, editor. Textbook of parasitology. 3rd ed, New York: Appleton-Century-Crofts; 1965. p. 1374.
17. Ash LR, Orihel TC, editors. Atlas of human parasitology. 2nd ed. Chicago: American Society of Clinical Pathologists Press; 1984. p. 212.
18. Yang J, Scholten T. A fixative for intestinal parasites permitting the use of concentration and permanent staining procedures. Am J Clin Pathol 1977;67:300-4.
19. Garcia LS, section editor. Parasitology. Section 7.1-7.4. In: Isenberg HD, editor. Clinical microbiology procedures handbook. Washington: American Society for Microbiology.
20. van Gool T, Mank TG. Fixatives and permanent stains in the laboratory diagnosis of intestinal protozoal infections. Haarlem, the Netherlands: Van Gool and Mank; 1999.
21. Vinjé J, Deijl H, van der Heide R, Lewes D, Hedlund K-O, Svensson L, et al. Molecular detection and epidemiology of Sapporo-like viruses. J Clin Microbiol 2000;38:530-6.
22. Abbink F, Duynhoven YTHP, de Wit M, Koopmans MPG, van Leeuwen WJ, Kortbeek LM. A population cohort study with a nested case-control study: a study design to estimate the incidence and aetiology of gastroenteritis (GE) in the Netherlands. Proceeding 4th World Congress of Foodborne Infections and Intoxications. 1998; Berlin, Germany. p. 766-9.
23. Sethi D, Wheeler J, Rodrigues LC, Fox S, Roderick P. Investigation of under-ascertainment in epidemiological studies based in general practice. Int J Epidemiol 1999;28:106-11.
24. Koopmans MPG, van Asperen IA. Epidemiology of rotavirus infections in The Netherlands. Acta Paediatr Suppl 1999;426:31-7.
25. Vinjé J, Altena SA, Koopmans MPG. The incidence and genetic variability of small round-structured viruses (SRSV) in outbreaks of gastroenteritis. J Infect Dis 1997;176:1374-8.
26. Borgdorff MW, Koopmans MPG, Goosen ESM, Sprenger MJW. Surveillance of gastroenteritis [letter]. Lancet 1995;346:842-3.

27. Wheeler JG, Sethi D, Cowden JM, Wall PG, Rodrigues LC, Tompkins DS, et al. Study of infectious intestinal disease in England: rates in the community, presenting to general practice, and reported to national surveillance. Br Med J 1999;318:1046-50.
28. Palmer S, Houston H, Lervy B, Ribeiro D, Thomas P. Problems in the diagnosis of foodborne infections in general practice. Epidemiol Infect 1996;117:479-84.
29. Nederlands Huisartsen Genootschap [Netherlands General Practitioners Society]. Standaard acute diarree. Huisarts en Wetenschap 1993;36(9).
30. Roderick P, Wheeler J, Cowden J, Sockett P, Skinner R, Mortimer P, et al. A pilot study of infectious intestinal disease in England. Epidemiol Infect 1995;114:277-88.
31. Monto AS, Koopman JS. The Tecumseh study: Occurrence of acute enteric illness in the community. J Epidemiol 1980;112:323-33.
32. Skirrow MB. A demographic survey of *Campylobacter, Salmonella* and *Shigella* infections in England. Epidemiol Infect 1987;99:647-57.
33. Simpson GE, Nagy GS, Fincks ES. A survey of acute gastroenteritis in general practice. Med J Aust 1969;2:633-5.
34. Giessen AW, van Leeuwen WJ, van Pelt W. Chapter 8: *Salmonella enterica* Serovar Enteritidis in the Netherlands: epidemiology, prevention and control. In: Saeed AM, editor. *Salmonella enterica* Serovar Enteritidis in humans and animals. Epidemiology, pathogenesis, and control. Ames (IA): Iowa State University Press;1999. p. 71-80.
35. Mank TG, Polderman AM, Zaat JOM, Roggeveen C, van Eijk JThM, Deelder AM. Persistent diarrhea in a general practice population in the Netherlands: prevalence of protozoal and other intestinal infections. In: Thesis intestinal protozoa and diarrhea in general practice. Haarlem, the Netherlands: Jos Mathôt BV; 1997. p. 47-64.
36. Tompkins DS, Hudson MJ, Smith HR, Eglin RP, Wheeler JG, Brett MM, et al. A study of infectious intestinal disease in England: microbiological findings in cases and controls. Commun Dis Public Health 1999;2:108-13.
37. Mank TG, Zaat JOM, Blotkamp J, Polderman AM. Comparison of fresh versus Sodium Acetate Acetic Acid Formalin preserved stool specimens for diagnosis of intestinal protozoal infections. Eur J Clin Microbiol Infect Dis 1995;14:1076-81.
38. de Wit MAS, Koopmans MPG, van der Blij JF, Duynhoven TYHP. Hospital admissions for rotavirus infection in the Netherlands. Clin Infect Dis 2000;31:698-704.
39. Mead PS, Slutsker L, Dietz V, McCaig LF, Bresee JS, Shapiro C, et al. Food-related illness and death in the United States. Emerg Infect Dis 1999;5:607-25.
